# Nanobodies Right in the Middle: Intrabodies as Toolbox to Visualize and Modulate Antigens in the Living Cell

**DOI:** 10.3390/biom10121701

**Published:** 2020-12-21

**Authors:** Teresa R. Wagner, Ulrich Rothbauer

**Affiliations:** 1 Pharmaceutical Biotechnology, Eberhard Karls University Tuebingen, 72076 Tuebingen, Germany; teresa.wagner@nmi.de; 2 Natural and Medical Sciences Institute, University of Tuebingen, 72770 Reutlingen, Germany

**Keywords:** nanobody, intrabody, phage display, live-cell imaging, biosensors, target validation

## Abstract

In biomedical research, there is an ongoing demand for new technologies to elucidate disease mechanisms and develop novel therapeutics. This requires comprehensive understanding of cellular processes and their pathophysiology based on reliable information on abundance, localization, post-translational modifications and dynamic interactions of cellular components. Traceable intracellular binding molecules provide new opportunities for real-time cellular diagnostics. Most prominently, intrabodies derived from antibody fragments of heavy-chain only antibodies of camelids (nanobodies) have emerged as highly versatile and attractive probes to study and manipulate antigens within the context of living cells. In this review, we provide an overview on the selection, delivery and usage of intrabodies to visualize and monitor cellular antigens in living cells and organisms. Additionally, we summarize recent advances in the development of intrabodies as cellular biosensors and their application to manipulate disease-related cellular processes. Finally, we highlight switchable intrabodies, which open entirely new possibilities for real-time cell-based diagnostics including live-cell imaging, target validation and generation of precisely controllable binding reagents for future therapeutic applications.

## 1. Selection of Intracellular Functional Nanobodies

More than thirty years after their discovery [1], antibody fragments derived from heavy-chain only antibodies of camelids, termed as variable heavy chain of heavy-chain only antibodies (VHH) or nanobodies (Nbs), have emerged as highly potent and versatile binding molecules for biomedical research, diagnostics and therapy [2]. With caplacizumab (ALX-0081), the first therapeutic Nb was approved in the U.S. and Europe for treatment of acquired thrombotic thrombocytopenic purpura in 2019 [3,4]. Compared to conventional antibodies (IgGs, ~150 kDa), Nbs are 10-times smaller (~15 kDa) and are characterized by stable folding, a compact structure, high solubility and chemical stability [2,5,6]. Due to their beneficial properties and accessibility to various cell compartments, Nbs applied as intrabodies have turned out to be highly suitable intracellular binding molecules (reviewed in [7,8,9]). Having the ability to specifically visualize and/or modulate endogenous targets within living cells, intrabodies provide distinct advantages compared to other technologies such as fluorescent fusion proteins, conditional gene expression, RNA interference and chemical or genetic knockouts. Nevertheless, when it comes to intracellular applications it has to be considered that Nbs, like any other antibody-derived binding molecule, comprise evolutionary conserved disulfide bonds. While the endoplasmic reticulum (ER) and mitochondria provide naturally suitable compartments for Nbs due to their (at least partially) oxidative environment, the reducing milieu of the cytosol strongly impairs formation of disulfide bonds. This can massively affect correct paratope formation and overall folding. Consequently, only Nbs whose functionalities do not rely on the formation of disulfide bonds are suitable candidates for the successful application as intrabodies. 

Considering that most Nbs yielded from conventional screening approaches are non-functional within living cells [10], multiple approaches have been developed to improve selection of intracellularly functional binders from synthetic or immunized libraries. One obvious strategy to overcome such limitations is to remove conserved cysteine residues within antibody-derived fragments. Therefore, either complete libraries or selected high affinity binders initially identified from conventional screenings such as phage display were site directly mutated to cysteine-free derivates [11]. However, in most cases, depletion of conserved disulfide bonds causes a major loss in stability, therefore extensive functional downstream analysis is needed, limiting throughput and diversity of intracellularly functional antibody fragments [11,12]. Alternatively, Olichon and Surrey genetically combined Nbs with the disulfide bond isomerase (DsbC), which catalyzes formation of disulfide bonds in the cytoplasm. Following this strategy, they were able to produce functional tubulin-binding Nbs in the cytoplasm of *E. coli* [13]. However, as this approach is restricted to certain expression systems and requires the generation of complex fusion proteins, it has some severe constraints which limit its broad application. Further studies suggested that, especially, physiochemical parameters have an important influence on the development of cytoplasmic stable intrabodies [14,15]. Recently, single chain antibody fragments (scFvs) were fused to peptide-tags, conferring a strong negative charge and low isoelectric point in order to generate so-called ultra-stable cytoplasmic antibodies (STANDs) [16]. In their study, the authors demonstrated how fusion of scFvs to negatively charged s3Flag and HA peptide-tags induces a significantly increased stability of aggregation-prone cytoplasmic intrabodies [16]. Notably, many Nbs are already strongly charged, showing either a positive or negative isoelectric point (pI) and therefore are less prone to aggregate at the most neutral pH of the cytoplasm [17]. In contrast to modifying sequences of existing Nbs, there is a long history of screening strategies aiming at initial identification of intrabodies. The first described methodology for this purpose was an adopted yeast two-hybrid (Y2H) assay [18]. For selection of bait/prey interactors, antibody fragments and antigens are fused to either a DNA-binding domain (bait) or to a transcriptional activation domain (prey). In the case of successful antigen binding, a reporter gene is activated and induces an easily detectable phenotype e.g., growth on selection media [19,20]. The advantage of this method is the flexible expression of potential intrabodies in cytosol and nucleus. However, it suffers from selecting large numbers of false positives and restricted screenable library sizes (>10^6^–10^7^ individual clones) [21]. Consequently, the intracellular antigen capture technology (IACT) was developed, in which a pre-selection step via phage-based biopanning is performed to enrich potential candidates prior to the Y2H screen [22,23]. Since its first description, the IACT has been successfully applied to identify consensus sequences within different binding molecules optimized for intracellular expression [24]. Thereby, a new selection strategy exclusively focusing on the complementarity-determining regions (CDRs) was established (iDabs) [22,25]. Similarly, Moutel et al. exploited the idea of intracellular optimized scaffolds. Based on a humanized synthetic Nb (hs2dAb) scaffold optimized for intracellular stability, they generated a fully synthetic phage display library of humanized Nbs from which they successfully selected effectively working intrabodies against a variety of targets including fluorescent proteins and cytoskeletal structures [10].

Other intrabody screening approaches employ a bacterial-two-hybrid (B2H) system specifically tailored for the identification of intrabodies based on distinct Nb scaffolds [21]. Compared to yeast, this bacterial-based system has clear advantages coming from faster growth rates and higher transformation efficiencies, two important characteristics that are beneficial for high-throughput selection of intrabodies. The B2H encompasses fusion constructs of Nbs and antigens with the lambda repressor and the α-subunit of the RNA polymerase, respectively. In the case of antigen binding within the bacterial cytoplasm, expression of selective marker genes essential for the survival under selection pressure is initiated and intracellular functional Nbs can be selected [21,26]. Notably, as the screening is performed in bacteria, functionality and specificity of B2H-selected intrabodies has to be further validated in eukaryotic systems e.g., by employing the mammalian cell-based fluorescent-2-hybrid (F2H) approach [27]. 

This limitation was elegantly overcome by Schmidt et al. using a lentiviral screening technology to select Nbs that are stably producible in mammalian cells and simultaneously neutralize influenza virus or vesicular stomatitis virus. For this approach, the Nb library derived from an immunized camelid was inserted into a lentiviral vector followed by the transduction of A549 cells. Upon application of a lethal dose of the virus of interest only those cells survived, which successfully expressed an effective viral neutralizing intrabody. In a proof-of-concept study, they described successful Nb selection and outlined a potential therapeutic application of virus-specific intrabodies [28]. Notably, similar concepts were realized by screening for functional scFv-derived intrabodies in mammalian cells. For example, scFv-derived intrabodies were selected by FACS analysis of impaired degranulation as a selection marker in rat leukemic cells [29], or intracellular binding of scFvs fused to the cytoplasmic domain of a receptor tyrosine kinase to its homo-oligomeric antigen became detectable by growth signal induction in a B-cell line [30]. Indisputably, high-throughput selection of target specific Nbs is challenging and the generation of stable, intracellularly functional Nbs adds another level of complexity to the screening process. However, as shortly outlined in this chapter, different screening approaches became available which support accelerated identification of suitable intrabodies based on Nbs. 

## 2. Delivery Systems of Intrabodies

After successful selection of intrabodies, the next challenge is to transport them into living cells or organisms. Here, one can chose between two options: transfer of the Nb-encoding cDNAs or introducing Nbs as purified proteins. While the first option is straightforward and employs broadly available and standardized expression systems in combination with effectively working transfection protocols e.g., using lipofection, electroporation or transfection of nanoparticles, these approaches suffer from substantial toxic side effects and hard-to-transfect cell types such as primary cells. Furthermore, expression levels do not directly correlate with the amount of transfected cDNA. Thus, resulting intrabody levels can be very heterogeneous and are hardly controllable. Therefore, while these approaches are easy applicable to studying intrabodies in single cells, they are less suitable for generation of intrabodies comprising cellular models which can be implemented e.g., in compound screening campaigns. Using a precise genomic insertion of intrabody-encoding sequences via CRISPR gene editing might be a more efficient approach to generate cell lines stably and homogenously expressing intrabodies [31]. Especially for in vivo analysis, gene delivery based on adeno-associated virus causing a long and stable expression, is preferred. For example, viral vector-based delivery of Nbs enabled live-cell STED microscopy of neuronal actin in mouse [32] or targeted degradation of α-synuclein in rats [33]. In consequence, combining cell-specific viral delivery with precise CRISPR gene editing could be an efficient way to introduce intrabodies into animal models for preclinical testing. Notably, gene delivery bears distinct advantages such as an easy guidance of intrabodies to defined cellular compartments by simply adding mitochondrial-, nuclear- or ER-localization and/or retention sequences [34]. However, current concerns regarding intrabodies transduced by gene delivery such as non-controllable expression yields, low cell or tissue specificity or unsolved safety issues still comprise major limitations for the development of intrabodies especially for therapeutic applications. 

To avoid genetic cell manipulation one might try to deliver intrabodies directly as proteins. Multiple methods and technologies for protein transduction are now available (reviewed in [35,36,37] with focus on antibodies) which also can be applied for Nbs. Physical methods including electroporation, microinjection, sonoporation or transfer by cell squeezing [38,39,40] can be used to transfer Nbs into cells. However, with these approaches only a limited number of cells can be addressed which afterward often suffer from severe cellular damage. Alternatively, Nbs can be coupled to protein transduction domains (PTDs) or cell-penetrating peptides (CPPs) to facilitate their cellular uptake. As most prominent examples, positively charged peptides such as HIV-1 TAT [41,42] have to be mentioned, but also other CCPs from natural and synthetic sources [43,44,45,46] have been successfully employed for cellular delivery of intrabodies. More recently, further approaches such as virus-like particles [47], silica nanoparticles [48], nanocapsules [49], polycationic resurfacing [50], charge-conversional polyion complex micelles [51], liposomal carriers [52] or oligoaminoamide carriers [53], to name a few, have been described as delivery options for Nbs. Notably, cellular transduction of Nbs has the exclusive advantage that any cargo in tow e.g., synthetic dyes or small molecule inhibitors, can be delivered alongside them. However, as holds true for other proteins, protein transduction still suffers from general low efficiencies, substantial mislocalization of the transduced Nb to endosomes and cellular toxicity. Since both methods, i.e., genetic- and protein-based intrabody delivery, still have substantial drawbacks especially for in vivo applications, further efforts have to be undertaken to improve cellular delivery of intrabodies before these molecules can be efficiently used in clinical testing. 

## 3. Intrabodies to Visualize Antigens in Living Cells

**Figure 1:**
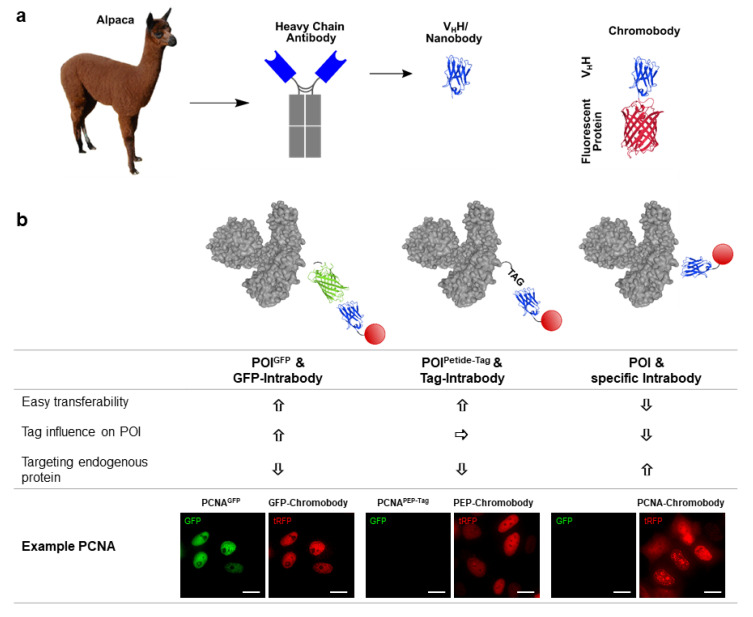
Intrabodies for visualization. (**a**) Schematic depiction of nano- and chromobodies derived from heavy-chain antibodies of *Camelidae* (**b**) Illustration of different labelling strategies for visualization of the protein of interest (POI) using intrabodies coupled to a detectable moiety and summary of their characteristics: POI genetically coupled to GFP recognized by a GFP intrabody, POI genetically coupled to a short peptide-tag recognized by the tag-specific intrabody and POI directly recognized by a specific intrabody. Model protein structures adapted from PDBs 3OGO, 5H88, 1EMA and 5LEW. Representative images of living Hela cells transiently expressing PNCA^GFP^ and the GFP-specific chromobody (tagRFP), PNCA^PEP-tag^ and the PEP-tag-specific chromobody (tagRFP) or PCNA-specific chromobody (tagRFP) as examples for the described labeling strategies are shown. Scale bar: 25 µm.

To visualize native cell structures and dynamic processes within living cells, imaging probes must not influence their target structures or intracellular processes. Thus, transiently binding intrabodies have become universal and versatile tools for live-cell imaging. Different strategies for how intrabodies have been used as imaging probes are summarized in the following (Figure 1). 

### 3.1. Chromobody Technology

To generate detectable intrabodies for live-cell imaging, Nbs have been genetically coupled to fluorescent proteins such as eGFP or RFP and introduced as DNA-encoded expression constructs in living cells. Reflecting their chimeric structure, those fluorescently labeled imaging probes were termed “chromobodies” (Cbs) [54] (Figure 1a). With ~40 kDa, Cbs are rather small and diffusive binding molecules that can access different cellular compartments. In contrast to genetic fusions or covalent labeling, intracellular antigen detection is distinguished by distinct on- and off-rates of Cbs, whereby the protein of interest (POI) is not constantly bound by the immuno-label. As a first example, a GFP-specific Cb was generated by labeling a GFP-binding Nb with mRFP1 followed by functional expression in the cytoplasm of mammalian cells. In a proof-of-concept study, real-time fluorescent co-localization analysis successfully visualized dynamic redistribution of GFP-tagged antigens in different cellular compartments following the GFP-Cb signal. Additionally, the detection of endogenous structures using Cbs against LaminA/C and human cytokeratin-8 paved the way to establish Cbs as novel and versatile probes for live-cell imaging [54]. 

Reflecting the broad accessibility of GFP-fusion proteins, a diverse range of toolkits using the GFP-Nb as an effectively working intrabody have been developed throughout the last decade. For example, GFP-Nb was integrated into different Nb-sensor fusions for detection and manipulation of a variety of intracellular dynamics. Thereby, a range of fluorescent sensors for Ca^2+^ (RGECO), H^+^ (SepH, pHuji) and ATP/ADP (PHR) or multimerization proteins were genetically coupled to the GFP-Nb and functionally applied in the cytoplasm, mitochondria and ER of mammalian cells [55]. Furthermore, GFP-Nb was implemented in sophisticated protein degradation mechanisms either generating complete knockouts or fine-tunable and inducible mechanisms controlling the degradation process [56,57,58,59]. Likewise, targeted gene induction was realized by employing GFP-Nbs in GFP-dependent transcription systems [60,61]. Notably, GFP-Nb has also been applied for in vivo studies e.g., as part of the so-called LlamaTag used to image transcription factors in fly embryos [62], or as a binding moiety to facilitate targeted protein mislocalization, summarized as FrabFP [63]. As extensively reported, the GFP-Nb/GFP-Cb system can be easily implemented in different applications and is continuously used for proof-of-principle studies demonstrating the suitability of intrabodies to manipulate cells and organisms. However, it suffers from the need of GFP-labeled POIs and as a consequence, the addition of a relatively large protein portion (~25 kDa) either to the N- or the C-terminus of the POI, which may considerably affect its expression level, activity and localization compared to endogenous counterparts [64,65,66].

### 3.2. Tag-Specific Intrabodies

Considering that shorter epitopes might have only a minor impact on native protein folding, function and protein–protein interactions, extensive efforts have been undertaken to identify Nbs specifically recognizing small peptide-tags. Although most Nbs preferably address conformational epitopes [2,67,68,69], more recently a handful of Nbs recognizing linear epitopes were identified, mostly as byproducts from screening campaigns against full-sized proteins. Some of these Nbs have been exploited as novel capture and detection systems. For purification of proteins comprising the small EPEA-tag (also known as C-tag) [70], Myc-tag or BC2- (Spot-) tag [71,72], Nbs were converted into affinity matrices such as the CaptureSelect^TM^ C-tag affinity matrix (www.thermofisher.com), the Myc-trap^©^ or Spot-trap^®^ resin (www.chromotek.com), respectively. 

Notably, beside their functionality to capture and detect peptide-tags, Nbs against the ALFA-tag [73], Moon-tag [74,75] and Pep-tag [76] have also been successfully applied as intrabodies. In the Cb format, these binders visualize cytoskeletal structures like vimentin and actin, nuclear structures like PCNA and mitochondrial outer membrane proteins such as Miro1 in live cells [73,76]. In addition, real-time single mRNA translation of ribosomes could be optically monitored in individual cells using the Moon-tag system. In combination with the Sun-tag based on a scFv-derived intrabody, start site selection of ribosomes was visualized, uncovering the extensive heterogeneity of mRNA decoding [75]. Such Cbs targeting protein- or peptide-tags substantially expanded the broad applicability of recombinant protein tagging. The development of different tag-specific intrabodies now further offers the possibility to visualize different cellular antigens in a multiplex manner within living cells simultaneously. Most importantly, the usage of universal tagging strategies in combination with well-defined intrabodies is a simple and easy adaptable option to study many different cellular POIs, as one does not need to generate intrabodies for each individual POI. However, it has to be considered that these approaches still rely on genetic manipulation and in most cases employ an artificial expression of tagged recombinant POIs. 

### 3.3. Intrabodies Targeting Endogenous Antigens 

In the light of the above-mentioned drawbacks of protein tagging, in some cases intrabodies targeting endogenous POIs are highly beneficial. Here, components of the cytoskeleton have to be especially mentioned. Due to the highly coordinated structure of the cytoskeletal network and its dynamic adaption to intra- and extracellular stimuli, even small interferences derived from the addition of large fluorescent protein-tags or covalently binding molecules can disturb their correct formation and dynamic relocalization dramatically [64,77,78,79]. To visualize these components in their native condition, a variety of Cbs have been developed. Prominent examples for cytoskeletal imaging probes are lamin-Cb [80], actin-Cb [81] or vimentin-Cb [82]. These Cbs have successfully been used e.g., to image compound-induced apoptosis by monitoring the integrity of the nuclear lamina [83], study nuclear actin in mammalian cells [84] or to trace induction of the epithelial–mesenchymal transition in response to external stimuli using vimentin as a cellular biomarker visualized by vimentin-specific Cbs [82,85]. Notably, actin-Cbs additionally visualized cytoskeleton remodeling in vivo e.g., during the different developmental stages in zebrafish embryos [81] or neuronal actin plasticity in mouse [32]. 

In combination with high-content real-time imaging, Cbs and Cb comprising cell models were further applied in screening campaigns to study compound effects on dynamic cellular processes tightly regulated by endogenous proteins. In this context, details of S phase progression and endogenous DNA replication was impressively revealed by using a PCNA-Cb [86] which also enabled high-throughput screening libraries for cell-cycle-affecting compounds [87]. Similarly, DNA-damaging agents were identified by monitoring the signal of a PARP1-Cb in live cells [88]. Moreover, histone-targeting Cbs visualized DNA-damaging sites upon detection of post-translational modifications in cells [89] or labelled chromatin structures in organisms [90]. To monitor real-time induction of the Wnt/β-catenin signaling pathway, which is highly important for embryonic development and is a key player in carcinogenesis, a Cb (BC1-Cb) which addresses cancer-related hypophosphorylated β-catenin was implemented in cellular models to study the effect of small molecules modulating the Wnt/β-catenin pathway [91,92]. 

To visualize the dynamics and native distribution of endogenous antigens, Cbs have to fulfil specific requirements: most importantly, they must not modulate the intrinsic function and localization of their antigen e.g., by binding catalytic sites or displacing central interaction partners. Furthermore, high-affinity Cbs can affect the dynamics and cellular distribution of their addressed antigens. In consequence, intracellular binding properties of Cbs have to be carefully analyzed. This can be addressed e.g., by targeted selection of Cbs addressing inert epitopes or selecting transiently binding Cbs, detectable by FRAP (fluorescent recovery after photobleaching) analysis [54,76,81,82]. Additionally, intracellular immune-precipitations (ICIPs) can be performed to analyze the proteome of Cbs [82,91,93]. In the case of stable Cb cell lines, these further require detailed phenotypic analysis evaluating their morphology, proliferation and signaling pathways in comparison to respective wild-type cells [82]. Notably, cellular Cb levels are only partially adjustable and the signal of bound Cbs can be outshined by the diffuse signal derived from non-bound Cbs. In this context, recently a phenomenon describing reduced proteasomal degradation of antigen-bound Cbs, summarized as antigen-mediated chromobody stabilization (AMCBS), was observed [93]. Recent studies further reveal how minor changes within the Cb sequence lead to turnover-accelerated [93], conditionally stable [94] or enhancer Cbs [95]. By implementation of these developments, Cbs have become more versatile imaging probes which not only visualize their endogenous antigens but can also be used to quantify changes in endogenous protein concentration in living cells [76,93]. 

### 3.4. Intrabodies as Biosensors

Considering that intracellular processes are highly complex and visualization is not always sufficient to study this their multifaceted nature, Nb-based biosensors have been developed which exhibit their “function” only after activation of the target protein. Such cellular biosensors have been successfully developed and applied e.g., to monitor activation of GPCRs in cellular models. In a pioneering work, Irannejad et al. generated a Cb (Nb80-GFP) specifically recognizing the conformational active form of the β_2_-adrenergic receptor (β_2_AR) in living cells. Upon activation of β2-AR with isoprenaline, this intracellular biosensor relocalizes from a diffuse fraction to the plasma membrane. Prolonged time-lapse imaging further revealed a displacement of the Cb to internalized β2-AR after binding of β-arrestin, followed by its redistribution to β2-AR-containing endocytic vesicles [96]. To generate biosensors against CCR7, the CDR1 and CDR3 regions of the Nb80 were randomized and three novel intrabodies specific for the cytoplasmic domain of CCR7 were selected from the synthetic Nb library. Upon fusion of these Nbs to split fragments of YFP and following the signal derived from bi-fluorescent complementation (BiFC), ligand-induced trafficking of CCR7 became detectable with high spatiotemporal resolution in living cells [97]. Similarly, intrabodies specific for active/agonist-bound state of opioid receptors (ORs) were identified, which monitored ligand-induced activation of ORs and revealed distortion of neuronal opioid receptor distribution caused by neuromodulating drugs [98]. Furthermore, via the RHO-Nb-GFP fusion protein, a BRET-based biosensor was developed, dynamically monitoring real-time RHO activation [99]. Another approach exploited Nbs as a diagnostic tool for sensing influenza A virus infection in a Nb-based sandwich reporter system. Via the nucleoprotein (NP)-dependent reporter gene transcription activation using NP-specific Nbs, infection of various influenza A subtypes in living cells has been monitored [100]. 

## 4. Intrabodies to Modulate and Manipulate Intracellular Antigens 

**Figure 2:**
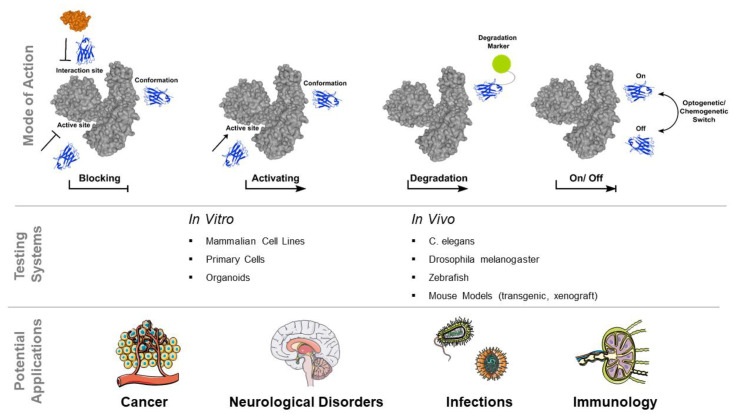
Intrabodies as intracellular modulators. Graphical representation how intrabodies can address their POI (protein of interest) for functional modulation. Shown are models of blocking and activating intrabodies, intrabodies inducing degradation and switchable intrabody systems. Additionally, commonly applied testing systems and potential clinical applications of intrabodies are listed. Model protein structures adapted from PDB 3OGO, 1EMA, 5LEW, 2EPE and parts of schematic art pieces are freely available from Servier Medical Art (https://smart.servier.com; Servier Medical Art by Servier is licensed under a Creative Commons Attribution 3.0 Unported License).

In addition to visualization, intrabodies can be selected to influence signaling pathways or function of cellular targets. Activating or blocking intrabodies have been developed for a range of POIs including enzymes, oncogenic proteins, proteins of the nervous system, virus proteins and toxins [101]. So far, many of those targets are still not druggable with small molecules. Therefore, intrabodies with high affinities and specificities are considered as interesting alternatives. In the following, a summary of promising intrabodies are depicted underlining their potential for preclinical research and clinical application (Figure 2).

### 4.1. Intrabodies in Oncology 

The most common strategies to modulate key drivers and intracellular signaling pathways in tumor therapy still rely on small molecules which often suffer from off-target binding which can cause severe side effects and overall toxicity [102,103]. Here, the specificity of intrabodies could be highly beneficial. A main driver in many cancer types is the deregulation of the tumor suppressor p53. By equipping an Nb binding the N-terminal transactivation domain of p53 with a mitochondrial localization signal, wild-type p53 was delocalized to mitochondria which affects the viability of tumor cell lines [104]. In contrast, a Nb targeting the DNA-binding domain of p53 was shown to stabilize endogenous p53 in a HPV-infected cervical cancer cell model [105]. In addition, GPCRs are well known to regulate a variety of cancer-associated signaling pathways like the PI3-protein kinase/AKT and MAPK pathway, therefore tight regulation of GPCRs is essential to block disease-related signaling. With Nb5, an intrabody which tightly binds to the Gβγ dimer and responds to all combinations of β- and γ subtypes competing with endogenous regulatory proteins, a control switch was identified. Due its potency to prevent Gβγ-mediated signaling events, this intrabody can be considered as a potential candidate for the treatment of different diseases driven by deregulated GPCRs [106]. In a similar context, in vitro studies were performed with protein kinase Cε modulating Nbs, either increasing or inhibiting its kinase activity on a cellular basis which therefore also might represent promising reagents for therapeutic applications [107]. Invasive and metastatic cancer types pointedly damaging surrounding tissues are non-operable and are therefore highly challenging. Consequently, advanced therapeutic approaches to stop cell migration and invasion are heavily needed. Recently, intrabodies targeting important regulators of formation of invadopodia such as fascin, cortactin and N-WASp (neural Wiskott–Aldrich syndrome protein) have been identified and applied in cellular live-cell studies. These studies showed that in the presence of such intrabodies, actin bundling can efficiently be disrupted in different cancer cell lines (including breast, prostate, head and neck cancer), hindering the formation of properly organized invadopodia [108,109,110,111]. Many of the examples mentioned above exhibit promising potential for cancer treatment *in cellulo*, however, before their maturation into clinical testing, these intrabodies have to prove their potency in living organisms. An encouraging example represents the F-actin caping protein (CapG)-specific Nb, which efficiently prevented migration of breast cancer metastasis in immune-deficient mice [112]. Another recently described STAT-3 binding intrabody suppresses the function of phosphorylated STAT-3 and distinctly reduces cell proliferation in vitro and breast cancer growth in mouse xenografts [113]. In summary, multiple Nb-based intrabodies are emerging as interesting candidates for the treatment of various malignancies, emphasizing the potential of these binding molecules for future clinical application in oncology. 

### 4.2. Intrabodies as Immune Modulators

According to the strong link between inflammation and development of malignancies, many intrabodies initially identified and analyzed in cancer models could also be applicable in the modulation of key players of immune signaling pathways. A classical target for immune modulators is the inflammasome, which in case of dysregulation causes a range of inflammatory diseases like arthritis, gout or diabetes. Therefore, obstruction of this innate immune regulator is highly interesting for many applications. A CARD (caspase activation and recruitment domain)-specific intrabody was shown to suppress assembly of the inflammasome which leads to a reduced secretion of proinflammatory cytokines. Furthermore, applied as Cb this intrabody visualized for the first time the filamentous structure of the adaptor protein ASC in living cells [114]. Additionally, activation of immune cells strongly relies on extensive cytoskeleton remodeling. Notable examples are podosome formation in macrophages and formation of an immunological synapse between T- and antigen-presenting cells. In this process, L-plastin, an actin-bundling protein specific to leukocytes, orchestrates assembly and turnover of actin filaments. De Clerque et al. showed that intrabodies trapping L-plastin in the active conformation induced T cell proliferation as well as cytokine secretion [115], whereas inhibiting intrabodies caused severe dysfunction of macrophages [116,117]. However, gelsolin-specific intrabodies applied in a similar approach showed no direct effect on podosome formation [116,118]. In summary, intrabodies manipulating immune-related targets offer new opportunities and advanced options exceeding current standards of care. 

### 4.3. Intrabodies to Address Neurological Disorders

Up to now, despite intensive research efforts, curative therapies are available only for a limited number of neurological disorders and neurodegenerative diseases. Thus, unconventional alternatives came into focus such as the application of intrabodies for the most prominent examples like Parkinson’s disease and Alzheimer’s disease [119]. Consequently, selection strategies have been adapted and validation pipelines for intrabody application have been developed to generate intrabodies specifically addressing targets in mammalian brain neurons [120]. Clearly, these developments are currently still in their infancy but encouraging examples are summarized in the following.

Actuators of many neurodegenerative diseases are mutated and misfolded proteins prone to aggregation continuously destroying parts of the brain and mental functions. In Parkinson’s disease, aggregated α-synuclein (α-syn) is the disruptive element and therefore the most promising target for therapeutic approaches. Ongoing research efforts including the application of computational affinity maturation [121] have led to the development of advanced α-syn intrabodies recognizing pathogenic aggregates at different stages of fibril maturation during disease progression [122] as well as post-translationally modified forms of α-syn [123]. The most promising Nb (NbSyn87) was linked to a PEST motif and induces a targeted degradation of α-syn aggregates in cellular models showing beneficial effects on proteostatic stress and cellular toxicity [124]. By this approach, the authors impressively demonstrated the general applicability of intrabodies fused to PEST domains for degradation of their respective antigens [124]. Moreover, upon viral vector-mediated delivery of this intrabody into a rat Parkinson’s disease model, an efficient removal of misfolded and aggregated α-syn in vivo was shown, turning this strategy into a highly interesting treatment option for synucleinopathies like Parkinson’s disease [33]. Regarding another aspect, oxidative stress-induced apoptosis in non-regenerative tissues such as the brain causes severe damage due to loss of functional cells. This cell death mechanism is associated with pathologies like Alzheimer’s and Parkinson’s disease. In this context, intrabodies targeting the pro-apoptotic protein Bax were demonstrated to block both mitochondrial membrane potential collapse and apoptosis after oxidative stress, bringing those tools into play as novel therapeutics [125]. Dysregulation of ion channel functions are also associated with several neurological and neuromuscular diseases. Many of these ion channels are directly regulated by GPCRs, therefore the Gβ-inhibiting intrabody identified by Gulati et al. also might be a potential candidate for therapy of Parkinson’s and Alzheimer’s disease or multiple sclerosis [106]. High-voltage-activated calcium channels (HVACCs) represent another class of ion channels important for neurological diseases like epilepsy, chronic pain and Parkinson’s disease. Here, HVACC-specific intrabodies in combination with an E3 ubiquitin ligase were successfully applied for targeted ion channel depletion. Expression of these intrabodies results in efficient current ablation, proving the potential of intrabodies to modulate ion channels [126]. To address vesicular glutamate transporters (VGLUTs) playing a key role in excitatory neurotransmission, intrabodies addressing the cytosolic domain of ratVGLUT1 have been developed and have been successfully shown to reduce the uptake of glutamate in reconstituted liposomes and subcellular fractions enriched with synaptic vesicles in vitro [127].

### 4.4. Intrabodies Inhibiting Viral and Bacterial Pathogens in Live Cells 

Considering intrabodies as inhibitors of viral or bacterial infections, human immunodeficiency virus (HIV) is the most prominent target when acknowledging the perpetual lack of a virus-depleting cure [128]. Up to now, a range intrabodies targeting different HIV-specific antigens including Rev [129,130], Vpr [131] and Nef [132,133] have been developed. Notably, Rev-specific intrabodies were shown to successfully inhibit viral replication and partly lower infectivity of viral particles as demonstrated in different cellular models [129,130]. With Nef intrabodies, even in vivo Nef-mediated effects could be reversed in transgenic mice, underlining the potential of intrabodies to fight against amplification and propagation of HIV in living organisms [132]. 

Due to mutational escape, the influenza virus represents a recurring seasonal predator. Therefore, in addition to vaccination, further possibilities to contain its spread are under development. Here, the nucleoprotein turned out to be an efficient target for generating intrabody-based inhibitors of influenza and a variety of different candidates were selected for disrupting virus replication in living cells [134,135]. Along this line, advanced treatment options fighting viral-induced hepatitis are urgently needed especially with the lack of efficient vaccinations e.g., for hepatitis virus C (HCV) which causes chronic infections with the risk of hepatocellular carcinoma. Several studies convincingly show that intrabodies targeting non-structural (NS) active proteins like NS3, NS4B and NS5B or the core protein, inhibit HCV replication [136,137,138]. Additionally, intrabodies addressing the core antigen of hepatitis B virus negatively affected expression and trafficking of the target antigen within live cells [139]. Facing an increasing number of antibiotic-resistant bacteria strains, a change of thinking towards alternative options is necessary. Thereby, intrabodies neutralizing bacterial toxins could become interesting for this field. Two promising examples of intrabodies addressing the SpvB toxin secreted by *Salmonella typhimurium* [140] and *Botulinum neurotoxin* [141] showed how such binders can rescue host cells and therefore function as potential antidotes. 

In summary, this chapter comprises only a short and non-comprehensive summary of recent findings on inhibitory intrabodies and their application in different preclinical disease models. Nevertheless, these developments impressively demonstrate how modulating intrabodies can successfully address a plethora of different cellular targets and contribute not only to a more detailed understanding of disease-related processes but also illustrate their potential for future therapeutic applications.

## 5. Switchable Intrabodies as Upcoming Tools 

A major disadvantage of intrabodies is their non-adjustable binding after expression. Therefore, switchable intrabodies are not only interesting for investigating cellular mechanisms but also open new opportunities for a controlled treatment which increases safety measurements substantially. Recently, different types of switchable systems were developed using light or small molecules as activators, termed as optogenetic or chemogenetic controls, respectively. Optogenetic switchable intrabodies were either realized by Nb fusions with light-responsive proteins [57,142] or by engineering light-controllable opto-intrabodies [143]. The first approach was developed for tunable light-induced depletion of target proteins and employs an E3 ubiquitin ligase fusion with CIBN (cryptochrome-interacting basic-helix-loop-helix 1 N-terminus) in combination with POI-specific intrabodies fused to Cryptochrome 2 (CRYP2). In the presence of short light pulses, conformational changes of the cryptochrome-associated proteins were triggered, thereby causing heterodimerization of both fusion proteins. Using this approach, intrabodies binding GFP, nuclear LaminA/C and PCNA were linked to the E3 ubiquitin ligase causing depletion of the respective target proteins [57]. Alternatively, to induce (switch on) intracellular binding, intrabodies targeting different POIs were split in two parts and fused to light-responsive proteins. Upon application of respective light-derived stimuli, their function was reactivated by bringing both halves together [142]. Similarly, Gil et al. engineered light-switchable Nbs by inserting the light-sensitive AsLOV2 domain into a solvent-exposed loop of an intrabody. Depending on the insertion position, light-induced binding or dissociation due to conformational changes was observed. With these developments a highly versatile photoswitchable nanobody toolbox enabling programmable regulation was established [143].

In contrast to light switchable systems, chemogenetic controllable systems bear the advantage of a stepwise regulation through precise titration of the active compound. Recently, such ligand-modulated binders have been generated by insertion of permutated bacterial dihydrofolate reductase (cpDHFR) in the CDR3 region of intrabodies. While the unstructured cpDHFR does not affect binding of the intrabody, addition of small compounds such as NADPH or trimethoprim changes the conformation of the cpDHFR, which results in the release of the bound antigen. This reversible process was nicely shown for several intrabodies inducing e.g., targeted antigen relocalization in living cells. It impressively demonstrates how intrabodies can be turned into switchable binders by the addition of small, non-toxic compounds [144]. Additionally, controlling protein function via switchable intrabodies in vivo is of great interest, thus providing the possibility of tunable phenotypes. This was successfully demonstrated by Deng et al., showing rapamycin-induced depletion of GFP-tagged CED-3 using the GFP-binding intrabody in combination with the E3 ubiquitin ligase, each fused to a rapamycin-responsive element. Following this strategy in a *C. elegans* model, the authors demonstrated how such an approach can be applied to influence e.g., induction of apoptosis in developing animals [57]. 

In summary, the field of Nbs validated for intracellular applications has widely increased over recent years, not only because of the ongoing development of advanced screening technologies for selection and generation of tailor-made intrabodies, but also due to their unique features and capabilities to monitor and manipulate cellular processes, thereby providing distinct advantages compared to conventional technologies such as fluorescent fusion proteins, siRNA, knock down genetics or chemical strategies using small molecules. Whereas non-modifying intrabodies for visualization purposes like Cbs have become state of the art versatile research tools, intrabodies for functional manipulation of disease-related antigens gain more and more importance for preclinical research. The most recent examples of intrabodies which modulate their target structures in living organisms using a switchable binding mechanism impressively provide a perspective on how intrabodies can be applied in advanced clinical research and can be implemented in novel therapeutic strategies, which are urgently needed for more personalized medicine in future.
